# Supplementary material for: Assessment of the diagnostic performance of the SD Bioline Malaria antigen test for the diagnosis of malaria in the Tombel health district, Southwest region of Cameroon
Source: PLoS One. 2025 Mar 13;20(3):e0298992. doi: 10.1371/journal.pone.0298992 (PMC11906078; doi:10.1371/journal.pone.0298992)
Supplement: S1 Data — (PDF) [file pone.0298992.s001.pdf]

Diagnostic test calculation

$$\text{Sensitivity} = \frac{\text{True positive}}{\text{True positive} + \text{False Negative}} \text{X } 100$$

$$\text{Specificity} = \frac{\text{False positive}}{\text{False negative} + \text{True positive}} \text{X } 100$$

$$\text{Positive Predictive Value (PPV)} = \frac{\text{True positive}}{\text{True positive} + \text{False Negative}} \text{X } 100$$

$$\text{Negative Predictive Value (NPV)} = \frac{\text{False Positive}}{\text{False Negative} + \text{True Positive}} \text{X } 100$$

$$\text{Accuracy} = \frac{\text{True Positive} + \text{False Negative}}{\text{True Positive} + \text{False Positive} + \text{False Negative} + \text{True Negative}} \text{X } 100$$

Measure	Formula (%)
Sensitivity	TP / (TP+FN)
Specificity	TN / (TN+FP)
PPV	TP / (TP+FP)
NPV	TN / (TN+FN)
Accuracy	TP+TN/ (TP+FP+FN+TN)

Were,

Measure	Formula (%)

Sensitivity	60 / (60+73)
Specificity	117 / (117+0)
PPV	60 / (60+0)
NPV	117 / (117+73)
Accuracy	60+117/ (60+0+73+117)

Confirmed by free online calculator (https://www.medcalc.org/calc/diagnostic_test.php, Version 23.0.8)

The calculator determines diagnostic test characteristics (sensitivity, specificity, PPV, NPV) and confidence intervals

**Test**	**Present**	**n**	**Absent**	**n**	**Total**
**Positive**	True Positive	a= 60	False Positive	c= 0	a + c = **60**
**Negative**	False Negative	b= 73	True Negative	d= 117	b + d = **190**
**Total**		a + b = **133**		c + d = **117**	

Disease prevalence

Enter: disease prevalence (%): 53.2

TEST

After a click on test, it will be computed and the result will appear automatically.

Results

**Statistic**	**Value**	**95% CI**
**Sensitivity**	45.11%	36.48% to 53.97%
**Specificity**	100.00%	96.90% to 100.00%
**Positive Likelihood Ratio**		
**Negative Likelihood Ratio**	0.55	0.47 to 0.64

**Disease prevalence (*)**	53.20%	
**Positive Predictive Value (*)**	100.00%	94.04% to 100.00%
**Negative Predictive Value (*)**	61.58%	57.87% to 65.15%
**Accuracy (*)**	70.80%	64.74% to 76.36%
