# Supplementary material for: Assessment of the diagnostic performance of the SD Bioline Malaria antigen test for the diagnosis of malaria in the Tombel health district, Southwest region of Cameroon
Source: PLoS One. 2025 Mar 13;20(3):e0298992. doi: 10.1371/journal.pone.0298992 (PMC11906078; doi:10.1371/journal.pone.0298992)
Supplement: S4 Data — (PDF) [file pone.0298992.s004.pdf]

Diagnostic test

Data

	Disease Present	Disease Absent	
Test Positive	60	0	60
Test Negative	73	117	190
	133	117	

Results

Sensitivity	45.113%	36.475% to 53.973%
Specificity	100.000%	96.896% to 100.000%
AUC	0.726	0.666 to 0.780
Positive Likelihood Ratio		
Negative Likelihood Ratio	0.549	0.470 to 0.640
Disease prevalence	53.200%	
Positive Predictive Value	100.000%	94.037% to 100.000%
Negative Predictive Value	61.579%	57.875% to 65.154%
Accuracy	70.800%	64.740% to 76.359%
