# Supplementary material for: Assessment of the diagnostic performance of the SD Bioline Malaria antigen test for the diagnosis of malaria in the Tombel health district, Southwest region of Cameroon
Source: PLoS One. 2025 Mar 13;20(3):e0298992. doi: 10.1371/journal.pone.0298992 (PMC11906078; doi:10.1371/journal.pone.0298992)
Supplement: S5 Data — (PDF) [file pone.0298992.s005.pdf]

```
ROC RDT BY MICROSCOPY (2)
  /PLOT=CURVE(REFERENCE)
  /PRINT= COORDINATES
  /CRITERIA=CUTOFF(INCLUDE) TESTPOS(LARGE) DISTRIBUTION(FREE) CI(95)
  /MISSING=EXCLUDE.
```

ROC Curve

Case Processing Summary

MP(Trophozoites/mm3)	Valid N (listwise)
Positive[a]	133
Negative	117

Larger values of the test result variable(s) indicate stronger evidence for a positive actual state.

a. The positive actual state is positive.

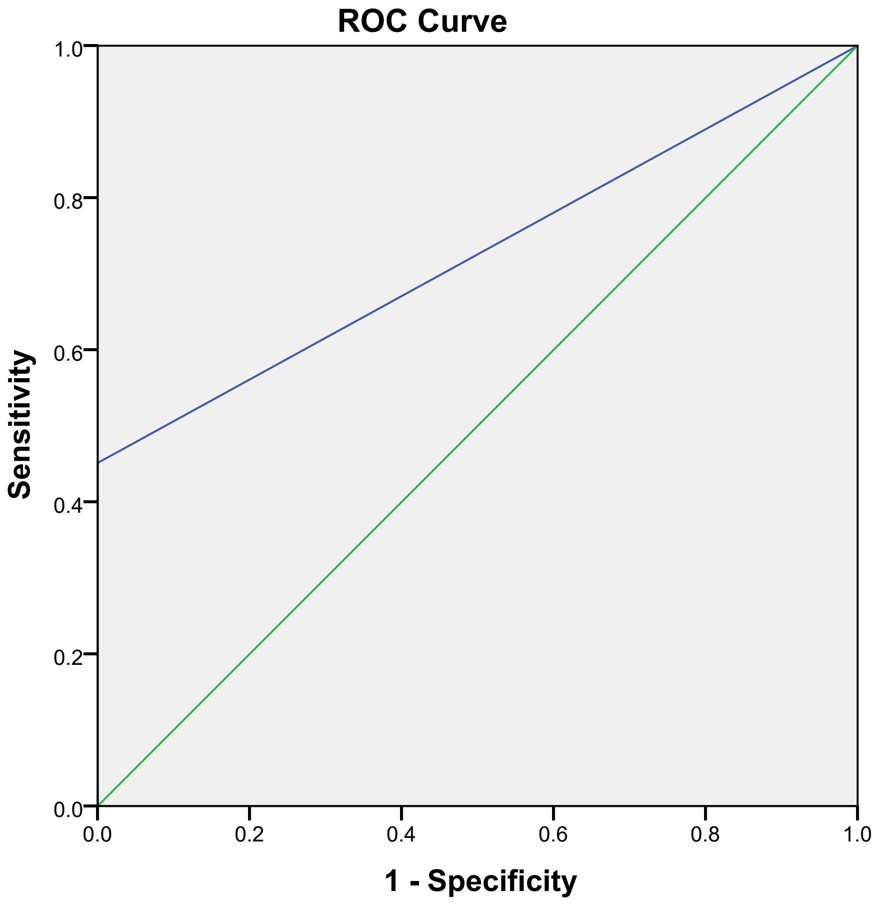

Diagonal segments are produced by ties.

Area Under the Curve

Test Result

Area
.726

The test result variable(s): RDT has at least one tie between the positive actual state group and the negative actual state group. Statistics may be biased.

Coordinates of the Curve

Test Result Variable(s): RDT

Positive if Greater Than or Equal To[a]	Sensitivity	1 - Specificity
.00	1.000	1.000
1.50	.451	.000
3.00	.000	.000

The test result variable(s): RDT has at least one tie between the positive actual state group and the negative actual state group.

a. The smallest cutoff value is the minimum observed test value minus 1, and the largest cutoff value is the maximum observed test value plus 1. All the other cutoff values are the averages of two consecutive ordered observed test values.

```
CORRELATIONS
  /VARIABLES=RDT MICROSCOPY
  /PRINT=TWOTAIL NOSIG
  /STATISTICS DESCRIPTIVES XPROD
  /MISSING=PAIRWISE.
```

Correlations

Descriptive Statistics

	Mean	Std. Deviation	N
RDT	1.24	.428	250
MP(Trophozoites/mm3)	1.53	.500	250

Correlations

		RDT	MP (Trophozoites/ mm3)
RDT	Pearson Correlation	1	.527**
	Sig. (2-tailed)		.000
	Sum of Squares and Cross-products	45.600	28.080
	Covariance	.183	.113
	N	250	250
MP(Trophozoites/mm3)	Pearson Correlation	.527**	1
	Sig. (2-tailed)	.000	
	Sum of Squares and Cross-products	28.080	62.244
	Covariance	.113	.250
	N	250	250

**. Correlation is significant at the 0.01 level (2-tailed).

```
NONPAR CORR
  /VARIABLES=RDT MICROSCOPY
  /PRINT=BOTH TWOTAIL NOSIG
  /MISSING=PAIRWISE.
```

Nonparametric Correlations

Correlations

			RDT	MP (Trophozoites/ mm3)
Kendall's tau_b	RDT	Correlation Coefficient	1.000	.527**
		Sig. (2-tailed)	.	.000
		N	250	250
	MP(Trophozoites/mm3)	Correlation Coefficient	.527**	1.000
		Sig. (2-tailed)	.000	.
		N	250	250
Spearman's rho	RDT	Correlation Coefficient	1.000	.527**
		Sig. (2-tailed)	.	.000
		N	250	250
	MP(Trophozoites/mm3)	Correlation Coefficient	.527**	1.000
		Sig. (2-tailed)	.000	.
		N	250	250

**. Correlation is significant at the 0.01 level (2-tailed).

```
CORRELATIONS
  /VARIABLES=RDT PD
  /PRINT=TWOTAIL NOSIG
  /STATISTICS DESCRIPTIVES XPROD
  /MISSING=PAIRWISE.
```

Correlations

Descriptive Statistics

	Mean	Std. Deviation	N
RDT	1.24	.428	250
Parasite densities	1.00	.967	250

Correlations

		RDT	Parasite densities
RDT	Pearson Correlation	1	.575**
	Sig. (2-tailed)		.000
	Sum of Squares and Cross-products	45.600	59.240
	Covariance	.183	.238
	N	250	250
Parasite densities	Pearson Correlation	.575**	1
	Sig. (2-tailed)	.000	
	Sum of Squares and Cross-products	59.240	232.996
	Covariance	.238	.936
	N	250	250

**. Correlation is significant at the 0.01 level (2-tailed).
