# Supplementary material for: Assessment of the diagnostic performance of the SD Bioline Malaria antigen test for the diagnosis of malaria in the Tombel health district, Southwest region of Cameroon
Source: PLoS One. 2025 Mar 13;20(3):e0298992. doi: 10.1371/journal.pone.0298992 (PMC11906078; doi:10.1371/journal.pone.0298992)
Supplement: S6 Data — (DOCX) [file pone.0298992.s006.docx]

**PROTOCOL**

**TITLE: Assessment of the diagnostic performance of the SD BIOLINE malaria antigen test for the diagnosis of malaria in the Tombel health district, Southwest region of Cameroon**

**Objective:** to assess the diagnostic performance the SD Bioline Malaria *Plasmodium falciparum* antigen rapid diagnostic test (Ag *P.f* RDT) for the diagnosis of malaria in the Tombel Health District (THD)

1. **Pre analytical phase**

- Recruitment of Participants
- Administration of questionnaire and Data Capture
- Conceptual framework

**Figure 1:** Conceptual framework for assessment of the diagnostic performance of the SD BIOLINE Malaria Ag Test.

1. **Data collection**

The data collection is done by using well-structured closed ended questionnaires. Confidentiality of participants will be guaranteed assured to the participants and questionnaires are administered in English.

1. **SD BIOLINE Malaria Antigen P.f HRP-II Rapid TEST**

**Aim:** to evaluate the diagnostic performance of the BIOLINE Malaria Antigen P.f HRP-II Rapid TEST.

**Principle**

The SD BIOLINE Malaria Antigen Pf Test contains a test trip coated with a mouse monoclonal antibody specific to Histidine-rich protein II (HRP-II). The letters T and C for “test line” and “control line” are on the surface of the test plate. The Line as well as the control Line are not visible in the results window before application of the samples. The control line is used to validate the process. The control line appears if the test is performed correctly and its reagents are working

**Equipment for mRDT**

1. An individually packaged test plate with desiccant
2. Test diluent
3. Single-used sample collection tool
4. Lancet/ Vacutainer’s syringe and butterfly needle and alcohol swab;
5. a pen and pencil and explanatory note

**Precaution and storage of the kit**

- The SD BIOLINE mRDT should not be stored in freezer
- The test plate is sensitive to humidity and heat
- Perform the test immediately after removing the plate from his bag
- Do not use after the expiration date
- The shelf life of the kit is indicated on the outer packaging
- Do not use the kit if the pouch is damaged or if it is not sealed
- When transporting and storing avoid exposure to high temperatures (above 45 degree) for more than one week.

The procedure of SD BIOLINE and microscopy are described according to a Ministry of Public Health manual book on National Malaria Control Program entitled: *Training manual for the capacity building of medical laboratory technicians on malaria diagnosis* in Cameroon; Oct 2021

**Procedure for RDT Test**

- Approximately 5μL of blood will be used to perform the test using the malaria Ag P.f RDT kit, following the manufacturer’s instructions.
- The mRDT cassette is label with participant identification and 5ul of blood was applied to the sample pad on the test strip.
- Immediately after the blood clotted, the applicator was firmly applied in the circle and two drops of buffer are applied vertically above the circle and left for 10 minutes to migrate after which the results were read.
- The results were read following the manufacturer’s instructions as: if a single pink colour appears on the control line “C”, the result was considered as negative.
- If a pink-coloured band appears on the “C” control line and a distinct pink coloured band also appears on Pf line, the result was considered as positive.
- The RDT results were obtained in the field by the principal investigator, a certified and experienced laboratory scientist.

1. **BIOLOGICAL DIAGNOSIS OF MALARIA**

**Equipment for microscopy**

1. Vacutainer’s syringe and butterfly needle will be used to collect venous blood;
2. Cotton wool will be used in making swabs to clean the collection site;
3. Alcohol will be used as disinfectant to clean the site for collection;
4. Gloves for safety measures;
5. Slides to hold objects for examination under the microscope;
6. a compound microscope fitted with paired 10x oculars (eyepieces), 10x, 40x and 100x objectives and a mechanical stage
7. a multiple tally counter or two-key tally counters, one to count malaria parasites and one to count white blood cells;
8. Giemsa-stained blood slides to be examined;
9. immersion oil, type A, high quality;
10. lens paper;
11. a pen and pencil and
12. a calculator.
13. **Thick blood film**

**Aim:** The tick film makes it possible to make biological diagnosis and quantify the parasitaemia. Unlike the thin smear, the thick film is more sensitive due to the concentration of blood on the slide.

**Principle:** the principle consists of depositing a drop of blood on a defibrinated object slide, dried and stained with Giemsa. The hemoglobin is dissolved and only remain visible under the microscope, the parasites, the platelets and white blood cells are also visible. Giemsa’s solution stains the cytoplasm of plasmodia blue while the component of chromatin of the nucleus is stained intense red.

**Procedure:**

- Note the date and the contact patient details of the patient on the slide
- Clean the area to prick preferably the palmar side of the end of 3 or 4 left finger with the cotton pad moistened with alcohol. Then let it dry for few second
- With the left hand, firmly press the proximal part of the cleaned finger to stimulate blood circulation and with a sterile vaccinostyle, prick with sharp and strong blow the pulp of the finger.
- Squeeze gently, and wipe again and collect 2 to 3 drops of blood with a dry cotton ball
- Cover the sting point with dry cotton
- With the corner of a second slide, quickly collect the drops of blood and spread them in a circular motion in spirals starting from the middle of drop to form a thick and uniform layer.
- The slides should be placed flat horizontally in a WHO type box to allow uniform drying away from dust, flies and heat for 45 minutes.

**Coloring with Giemsa:**

- Giemsa stock solution concentrate, staining tray, buffered water pH = 7.2 or distilled water, rack and graduated cylinders to different sizes.

**Operating mode**

- Prepare a 10% Giemsa solution (10ml of Giemsa stock solution in 90 ml of buffered water at 7.2 pH / distilled water). One volume of Giemsa stock solution in 9 volumes of buffered water
- Place the slides back-to-back in the staining tank
- Slowly pour the solution until the slides are completely immersed and leave to stand for 10 to 15 minutes, staining away from sunlight
- Gently remove the solution by slowly adding clean water, evacuate the colorant foam deposit on the tank then rinse the slides
- Remove the slides one by one and let them drain and dry on a rack, with the surfaces bearing the samples facing down

**Reading with an optical microscope**

- Place the slide on the stage and focus with the 10x objective and possibly see other blood parasites
- Put a drop of oil on the thick film, then go to the 100x objective and make sure that: the lamp rheostat is fully open, the capacitor raised, the iris diaphragm fully open, ensure that the chosen area has the required quality and examine the thick film on at least 100 microscopic fields using the rampart method

**Performing a parasite count on a thick film**

- Parasite density (Parasite/µL of blood) are and counted calculated using the WHO recommended assumed WBC count of 8000µL of blood
- Place the glass slide on the microscope stage with the label to the left. This allows a standardized approach for the start point for counting and also to record parasite locations using the marked divisions on the slide holder.
- The number of leucocytes counted varies between 200 and 500 according to the following scheme: If you have counted ≥ 100 parasites in 200 white cells, stop counting, and record the results as the number of parasites per 200 white cells. If you have counted ≤ 99 parasites in 500 white cells, stop counting, and record the results as the number of parasites per 500 white cells. However, if on the other hand, at 200 leucocytes, the number of parasites counted is less than 100, then it is necessary to continue up to 500 leucocytes.

**Calculating of parasites density**

- Parasite density= (number of asexual parasite counted)/ (WBC counted) X 8000/µL blood.
- Parasite densities are used according to the estimation parasite densities reported by Kosack et al [20] as low density = 10 to 90 parasites/μl; moderate density = 100 to 1,000 parasites/μl, and high density = ˃ 1,000 parasites/μl.

1. **Thin smear**

**Principle:** the thin smear is a technique which makes it possible to recognize species more quickly and easily than to the observation of morphology of parasite and of the parasitized erythrocyte. It requires fixation with methanol, followed by staining with Giemsa

**Material and reagents (see thick film)**

**Operating mode**

- Note the date and contact details of the participant (number) on the slide
- Clean the place of prick (preferably the palmar side of the end of 3 to 4 left finger) with a cotton pad moistened with alcohol. Then let it dry for few seconds
- With the left hand, firmly press the proximal part of the cleaned finger to stimulate the circulation and with a sterile vaccinostyle, prick with a sharp and strong blow the pulp the finger
- Squeeze gently, and wipe off the first drop of blood with dry cotton
- Place 2µl of blood on one edge of a slide
- Place the edge of a second clean object slide in front of the drop of blood and slide it black until it comes into contact with the drop of blood which will spread over the entire width of it
- Tilt the blade at an angle of 45degree.
- Slide it with a rapid and regular movement, forward towards the free end of the object slide carrying the drop of blood
- The thin film must have two edges and a tail, elective reading areas
- **Thin film staining technique** (same with thick film plus methanol)
- **Operating mode**: fix the thin film with methanol
- **Staining in the same way as thick film**
- **Morphological characters of different plasmodia species:** the morphology of the plasmodia species such as the shape, size and staining characteristics are described according to a Ministry of Public Health manual book on National Malaria Control Program entitled: *training manual for the capacity building of medical laboratory technicians on malaria diagnosis* in Cameroon; Oct 2021; Pages 19-24. And to WHO Standard Operating Procedure (SOP -08): Microscopy examination of thick and thin blood films for identification of malaria parasites),

**Waste managements:**

Waste was separated into infectious, non-infectious and sharps. The infectious were placed in separate bag from the non-infectious and the sharps kept in a closed container. All the waste was disposed through the process of incineration by the cleaner.
