# Supplementary material for: Assessment of the diagnostic performance of the SD Bioline Malaria antigen test for the diagnosis of malaria in the Tombel health district, Southwest region of Cameroon
Source: PLoS One. 2025 Mar 13;20(3):e0298992. doi: 10.1371/journal.pone.0298992 (PMC11906078; doi:10.1371/journal.pone.0298992)
Supplement: Attachment — Submitted filename: PONE-D-23-43663_reviewer.pdf [file pone.0298992.s008.pdf]

PLOS ONE

Assessment of the diagnostic performance of the SD Bioline malaria antigen Plasmodium falciparum rapid diagnostic test for the diagnosis of malaria in the Tombel health area, Southwest region of Cameroon

--Manuscript Draft--

**Manuscript Number:**	PONE-D-23-43663
**Article Type:**	Research Article
**Full Title:**	Assessment of the diagnostic performance of the SD Bioline malaria antigen Plasmodium falciparum rapid diagnostic test for the diagnosis of malaria in the Tombel health area, Southwest region of Cameroon
**Short Title:**	RDT for the diagnosis of malaria in the Tombel health area, Southwest region of Cameroon
**Corresponding Author:**	Watching Djakissam, Ph.D<br>University of Buea<br>BUEA, CAMEROON
**Keywords:**	malaria; RDT; validity; accuracy; Microscopy, Cameroon.
**Abstract:**	Background Rapid diagnostic tests (RDTs) are commonly used for the diagnosis of malaria in resource-limited settings. However, the accuracy of RDTs may vary depending on the population being tested and the prevalence of malaria in the study area.<br>Objective The aim of this study was to assess the diagnostic performance Ag P.f RDT for the diagnosis of malaria in the Tombel Health Area (THA).<br>Materials and Methods This was a cross-sectional community-based study, which targeted persons living within the THA and was conducted from March to May 2023. A consecutive sampling technique was used to collect blood samples of 250 individuals and tested using the SD Bioline Malaria Ag P.f RDT and microscopy. Data was analyzed using SPSS version 25.0. The sensitivity, specificity, positive predictive value (PPV), negative predictive value (NPV) were calculated to assess the diagnostic accuracy of the RDT.<br>Results Microscopy identified 133 (53.2%) participants with malaria and the mRDT identified 60 cases. The sensitivity of Malaria Ag P.f RDT was 45.0% (95%CI: 37-45), the test correctly identified 60 (45%) of true positive P. falciparium cases. The specificity of 100% (95%CI: 96-100) showed that the test correctly identified all negative cases. PPV was 100% (95%CI: 94-100), there is a very high likelihood that all the positive cases are truly infected. The lower NPV of 61.5% (95%CI: 54-68), there is still a chance the negative cases might be infected. The AUC = 0.78 with moderate diagnostic suggesting that there is 22% chance that the test can produce a false result.<br>Conclusion The overall accuracy of P.f HRP2 in the THA is moderate. This level of accuracy may be acceptable in some contexts, but it is not ideal for a diagnostic test, particularly for a disease like malaria that can have serious health consequences if left untreated.
**Order of Authors:**	Ngalame Abigail Lumateh, MSc
	Watching Djakissam, Ph.D
	Odette Dzemo Kibu, Ph.D
	Elisabeth Menkem Zeuko'o, Ph.D
	Dickson SHEY NSAGHA
**Additional Information:**	
**Question**	**Response**
**Financial Disclosure**<br><br>Enter a financial disclosure statement that describes the sources of funding for the	The author(s) received no specific funding for this work.

Powered by Editorial Manager® and ProduXion Manager® from Aries Systems Corporation

work included in this submission. Review the submission guidelines for detailed requirements. View published research articles from *PLOS ONE* for specific examples.<br><br>This statement is required for submission and **will appear in the published article** if the submission is accepted. Please make sure it is accurate.<br><br>**Funded studies**<br>Enter a statement with the following details:<br>• Initials of the authors who received each award<br>• Grant numbers awarded to each author<br>• The full name of each funder<br>• URL of each funder website<br>• Did the sponsors or funders play any role in the study design, data collection and analysis, decision to publish, or preparation of the manuscript?<br><br>Did you receive funding for this work?	
**Competing Interests**<br><br>Use the instructions below to enter a competing interest statement for this submission. On behalf of all authors, disclose any competing interests that could be perceived to bias this work—acknowledging all financial support and any other relevant financial or non-financial competing interests.<br><br>This statement is required for submission and **will appear in the published article** if the submission is accepted. Please make sure it is accurate and that any funding sources listed in your Funding Information later in the submission form are also declared in your Financial Disclosure statement.<br><br>View published research articles from *PLOS ONE* for specific examples.	The authors have declared that no competing interests exist.

**NO authors have competing interests**<br><br>Enter: *The authors have declared that no competing interests exist.*<br><br>**Authors with competing interests**<br><br>Enter competing interest details beginning with this statement:<br><br>*I have read the journal's policy and the authors of this manuscript have the following competing interests: [insert competing interests here]*<br><br>* typeset	
**Ethics Statement**<br><br>Enter an ethics statement for this submission. This statement is required if the study involved:<br><br>• Human participants<br>• Human specimens or tissue<br>• Vertebrate animals or cephalopods<br>• Vertebrate embryos or tissues<br>• Field research<br><br>Write "N/A" if the submission does not require an ethics statement.<br><br>General guidance is provided below. Consult the submission guidelines for detailed instructions. **Make sure that all information entered here is included in the Methods section of the manuscript.**	Ethical Clearance was obtained from the Ethical Committee Commission from the Institutional Review Board (FHS-IRB reference number: 2023/1992-02/UB/SG/IRB/FHS) of the University of Buea. Administrative authorizations were obtained from the Department of Public Health, Faculty of Health Sciences, South West Regional Delegation of Public Health (SW-RDPH) (reference number: R11/MINISANTE/SWR/RDPH/PS/544/576) and Tombel District Hospital before data collection. Informed consent was obtained from the participants. Participant's information was obtained and kept confidentially by not taking any of their personal information such as names; rather codes were assigned to each participant after data collection and each participant was given equal treatment.

Powered by Editorial Manager® and ProduXion Manager® from Aries Systems Corporation

**Format for specific study types**<br><br>**Human Subject Research (involving human participants and/or tissue)**<br>• Give the name of the institutional review board or ethics committee that approved the study<br>• Include the approval number and/or a statement indicating approval of this research<br>• Indicate the form of consent obtained (written/oral) or the reason that consent was not obtained (e.g. the data were analyzed anonymously)<br><br>**Animal Research (involving vertebrate animals, embryos or tissues)**<br>• Provide the name of the Institutional Animal Care and Use Committee (IACUC) or other relevant ethics board that reviewed the study protocol, and indicate whether they approved this research or granted a formal waiver of ethical approval<br>• Include an approval number if one was obtained<br>• If the study involved *non-human primates*, add *additional details* about animal welfare and steps taken to ameliorate suffering<br>• If anesthesia, euthanasia, or any kind of animal sacrifice is part of the study, include briefly which substances and/or methods were applied<br><br>**Field Research**<br><br>Include the following details if this study involves the collection of plant, animal, or other materials from a natural setting:<br>• Field permit number<br>• Name of the institution or relevant body that granted permission	
**Data Availability**<br><br>Authors are required to make all data underlying the findings described fully available, without restriction, and from the time of publication. PLOS allows rare exceptions to address legal and ethical concerns. See the PLOS Data Policy and FAQ for detailed information.	Yes - all data are fully available without restriction

Powered by Editorial Manager® and ProduXion Manager® from Aries Systems Corporation

A Data Availability Statement describing where the data can be found is required at submission. Your answers to this question constitute the Data Availability Statement and **will be published in the article**, if accepted.<br><br>**Important:** Stating 'data available on request from the author' is not sufficient. If your data are only available upon request, select 'No' for the first question and explain your exceptional situation in the text box.<br><br>Do the authors confirm that all data underlying the findings described in their manuscript are fully available without restriction?	
**Describe where the data may be found in full sentences. If you are copying our sample text, replace any instances of XXX with the appropriate details.**<br><br>• If the data are **held or will be held in a public repository**, include URLs, accession numbers or DOIs. If this information will only be available after acceptance, indicate this by ticking the box below. For example: *All XXX files are available from the XXX database (accession number(s) XXX, XXX.).*<br>• If the data are all contained **within the manuscript and/or Supporting Information files**, enter the following: *All relevant data are within the manuscript and its Supporting Information files.*<br>• If neither of these applies but you are able to provide **details of access elsewhere**, with or without limitations, please do so. For example:<br><br>*Data cannot be shared publicly because of [XXX]. Data are available from the XXX Institutional Data Access / Ethics Committee (contact via XXX) for researchers who meet the criteria for access to confidential data.*<br><br>*The data underlying the results presented in the study are available from (include the name of the third party*	All relevant data are within the manuscript and its Supporting Information files

and contact information or URL).<br>• This text is appropriate if the data are owned by a third party and authors do not have permission to share the data.<br><br>* typeset	
Additional data availability information:	Tick here if your circumstances are not covered by the questions above and you need the journal's help to make your data available.

Powered by Editorial Manager® and ProduXion Manager® from Aries Systems Corporation

Manuscript Click here to access/download;Manuscript;Ngalame et al Manuscript revised March 2024.doc

Assessment of the diagnostic performance of the SD Bioline malaria antigen Plasmodium falciparum rapid diagnostic test for the diagnosis of malaria in the Tombel health area, Southwest region of Cameroon

Abigail L Ngalame[1¥], Watching Djakissam[2¥], Odette Z. Kibu [1&], Elisabeth M. Zeuko'o [3&], Dickson S. Nsagha [1¥]

1: Department of Public Health and Hygiene, Faculty of Health Sciences, University of Buea P.O Box 63, Buea, South West Region of Cameroon.

2: Department of Medical Laboratory Sciences, Faculty of Health Sciences, University of Buea P.O Box 63, Buea, South West Region of Cameroon.

3 Department of Biomedical Sciences, Faculty of Health Sciences, University of Buea P.O Box 63, Buea, South West Region of Cameroon.

*[a] Current Address: Department of Public Health and Hygiene, Faculty of Health Sciences, University of Buea P.O Box 63, Buea, South West Region of Cameroon.

*[b] Current Address: Department of Medical Laboratory Sciences, Faculty of Health Sciences, University of Buea P.O Box 63, Buea, South West Region of Cameroon.

Corresponding authors

1. Email: nsaghads@hotmail.com (DSN)

2. E-mail: djakissamwatching@yahoo.fr (WD)

[¥]These authors contributed equally to this work.

[&]These authors also contributed equally to this work.

ABSTRACT

Background Rapid diagnostic tests (RDTs) are commonly used for the diagnosis of malaria in resource-limited settings. However, the sensitivity of RDTs may vary depending on the brand being used

Objective The aim of this study was to assess the diagnostic performance Ag *P.f* RDT for the diagnosis of malaria in the Tombel Health Area (THA).

Materials and Methods This was a cross-sectional community-based study targeting persons living within the THA during March to May 2023. A consecutive sampling technique was used to collect blood samples of 250 individuals and tested using the SD Bioline Malaria Ag *P.f* RDT and microscopy. Data was analysed using SPSS version 25.0. The sensitivity, specificity, positive predictive value (PPV) and negative predictive value (NPV) were calculated to assess the diagnostic accuracy of the RDT.

Results Microscopy identified 133 (53.2%) participants with malaria and the mRDT identified 60 cases. The sensitivity of Malaria Ag *P.f* RDT was 45.0% (95%CI: 37-45), the test correctly identified 60 (45%) of true positive *P. falciparium* cases. The specificity of 100% (95%CI: 96-100) showed that the test correctly identified all negative cases. PPV was 100% (95%CI: 94-100), there is a very high likelihood that all the positive cases are truly infected. The lower NPV of 61.5% (95%CI: 54-68), there is still a chance the negative cases might be infected. The AUC = 0.78 with moderate diagnostic suggesting that there is 22% chance that the test can produce a false result.

Conclusion The overall accuracy of *P.f* HRP2 in the THA is moderate. This level of accuracy may be acceptable in some contexts, but it is not ideal for a diagnostic test,

particularly for a disease like malaria that can have serious health consequences if left untreated.

Keywords: Malaria; RDT; validity; accuracy; Microscopy, Cameroon.

INTRODUCTION

Malaria continues to be a serious global public health concern. The World Health Organization (WHO) reported 405,000 malaria deaths and 228 million cases in 2018 worldwide [1]. Furthermore, 247 million cases of malaria were reported in 2021, 245 million cases in 2020, according to the World Malaria Report [2]. Compared to 625,000 in 2020, the expected number of malaria deaths in 2021 was 619 000. In Sub-Saharan Africa (SSA), the figures for cases and fatalities were 93% and 94% respectively. The risk of malaria transmission remains high throughout the whole country of Cameroon, making it a severe public health concern. Malaria accounts for 22% of annual mortality in Cameroon and is the leading cause of hospitalization (46%) and consultation (26%). Over 95% of cases are caused by the primary pathogen, *Plasmodium falciparum* [5].

The most potent antimalarial drugs is artemisinin-based combination therapy (ACTs), WHO advises treating a patient only if a test for malaria is positive, since there won't be a viable substitute for artemisinin derivatives for a number of years. This is necessary to maintain the effectiveness of ACTs. Effective malaria management requires a timely and precise diagnosis. In Cameroon, clinical assessment, microscopy, and rapid diagnostic tests (RDTs) are the main methods used to diagnose malaria [7]. Microscopy has significant limitations that prevent its use in resource-constrained nations like Cameroon, where the prevalence of malaria is highest. These include a lengthy return time, an unstable power supply, and the requirement for technical competence. Consequently, RDT was created to increase the objectivity and sensitivity of diagnosing malaria without entirely depending on microscopy [8]. RDT performance, however, can be impacted by a

number of factors, such as the product's format and type, storage conditions, short shelf life due to temperature instability, particularly during transportation from the manufacturing or supply location to the final place of use, suboptimal sensitivity at low parasite densities, and the inability to identify the species of the parasite or quantify its density [9,10,11, 12]. Inaccurate diagnostic testing can result in incorrect therapy, raising the possibility of morbidity and death [13]. Consequently, a cross-sectional community-based investigation is required to evaluate the diagnostic performance of these RDT for the diagnosis of malaria in rural populations. This research will yield important data regarding the precision of the RDT utilized in the THA as well as its potential benefits for enhancing malaria diagnosis and treatment in rural settings [14]. Therefore, this study was designed to assess the diagnostic performance of the SD BIOLINE malaria Antigen (Ag) *Plasmodium falciparum* (*P.f*) test for the diagnosis of malaria in the Tombel Health Area, Southwest region of Cameroon.

MATERIALS AND METHODS

Study Design

This was community based cross-sectional study carried out in the Tombel Health Area in the South West Region of Cameroon.

Study Period

Protocol was developed, data collected, analysed and interpreted within a period of 6 months, that is from February to July 2023. Data were collected and tested simultaneously all over the investigation period. Administration of questionnaire and collection of samples started on the 30th of April 2023 and was ended on the 20th of June 2023.

Study area and setting

Tombel Health District is a health district located in the Kupe-Muanenguba Division of the South West Region of Cameroon. There are seven health areas found in the Tombel Health District including Tombel, Baseng, Ebonji, Nyasoso, Edibenjock, Ndom and Ndibejock. The THD is situated in a rural area, with a population of approximately 65000 people. The major sociological groups are Bakossi, Grass landers, Nigerians, Banyangi, Oroko. In general, the population consists of -Approximately 35,690 males – Approximately 28,587 females– And about 24 901 children aged 0-16 years. The health area is characterized by dense forests, hilly terrains, and a warm and humid climate. There are two main seasons; long rainy (8-9 months) and short dry (3-4 months) seasons [15].

The health indicators in the THA are generally poor, a high prevalence of infectious diseases such as malaria which affects all age group. This is because they are surrounded by bushes and also carryout farming activities that exposes them to the causative agent of the disease regularly [15]. However, efforts are being made to improve health outcomes in the area, including the provision of training and support for health workers, the expansion of health facilities, and the implementation of community-based health programs.

Tombel Health Area (THA) made up of approximately 9000 persons divided in about 9 quarters is served by a government hospital, several health centres, and private clinics. The government hospital is the Tombel District Hospital, which is a secondary-level facility with a capacity of about 30 beds. The hospital provides general medical services, including outpatient and inpatient care, maternal and child health, laboratory services, and basic surgical procedures.

There are also several health centres located throughout the health area that provide primary health care services, including immunizations, family planning, antenatal care,

and treatment of common illnesses. These health centres are staffed by trained nurses and midwives.

Despite the presence of health facilities, access to health care remains a challenge in Tombel Health Area due to the rugged terrain and poor road network. Additionally, the health facilities face challenges such as inadequate staffing, limited medical supplies, and a lack of equipment and infrastructure.

Study population

All persons living in the Tombel Health Area aged from 5 years and above.

Inclusion criteria

Persons living in the Tombel Health Area with or without fever at the time of recruitment

Persons who had not had antimalarial within the last two weeks at the time of recruitment.

Exclusion criteria

Persons on antimalarial or medications like doxycycline that affect malaria parasitaemia.

Persons who visited from different health areas for consultation**.**

Sample size determination

We used the sample size calculation proportions of sensitivity and specificity method of Burderer and collaborators 1996 [16]

$$n = n1 + n2$$

n1=(TP+FN)/P

n2=(TN+FP)/(1-P)

And

TP+FN=Z^2 (Sensitivity(1-Sensitivity))/W^2

TN+FP=Z^2 (Specificity(1-Specificity))/W^2.

Where,

n_1 and n_2 = required number of participants for sensitivity and specificity with estimated sensitivity and specificity of 95% and 99% respectively [17].

P = prevalence of malaria from previous study (34.7%) [18].

Z = standard normal variation at a confidence level of 95% (standard value of 1.96)

W = 0.05 (10% maximum acceptable width of the 95% confidence interval)

Substituting in the equation above, n_1 required for sensitivity = 215 participants n_2 required for specificity = 24 participants at least n = 239 participants were used for this study.

Sampling technics

A consecutive sampling technique was used, based on the quarters in the THA. Probability proportionate to size was used to get number of participants needed from each quarter in the THA as shown in table 1. Within each quarter, participants were selected consecutively until the expected number of participants allocated for each quarter was attained.

Table 1: Probability proportionate to size sampling of participants in various quarters

Residence	No (%)	Proportionate sample size
**CDC Road**	360(4.0)	13
**Ebubu**	288(3.2)	10
**Elom**	180(2.0)	6
**KUPE**	756 (8.4)	22
**Peng**	252(2.8)	8
**Mile19**	540(6.0)	16
**Mile20**	360(4.0)	11
**NGAB**	396(4.4)	12
**WARDS**	5868(65.2)	170
**TOTAL**	**900**	**260**

Study procedure

Ethical consideration

Ethical Clearance was obtained from the Ethical Committee Commission from the Institutional Review Board (FHS-IRB reference number: 2023/1992-02/UB/SG/IRB/FHS) of the University of Buea. Administrative authorizations were obtained from the Department of Public Health, Faculty of Health Sciences, South West Regional Delegation of Public Health (SW-RDPH) (reference number: R11/MINISANTE/SWR/RDPH/PS/544/576) and Tombel District Hospital before data collection. Informed consent was obtained from the participants. Participant's information was obtained and kept confidentially by not taking any of their personal information such as names; rather codes were assigned to each participant after data collection and each participant was given equal treatment.

Pre analytical phase

Recruitment of Participants

Participants were informed about the research and its objectives. Those who accepted to sign the consent forms answered the questionnaires.

Administration of questionnaire and Data Capture

A structured questionnaire was administered to each of the participant after they signed the consent forms for the major participants and assent forms by the parent or guardians for minor participants. Socio-demographic data such as age, gender, occupation, marital status was obtained.

Data collection

The data collection was done by using well-structured closed ended questionnaires. Confidentiality of participants was guaranteed assured to the participants and

questionnaires were administered in English. These questionnaires were used to get information on the socio-demographic characteristics of the participants as well as the clinical diagnosis. Blood samples were collected by pricking the finger of each participant and using the sample to conduct a Rapid Diagnostic Test and a thin and thick film for microscopy.

Laboratory investigations

Thick film:

The slides were labelled with the participant's unique identification number. A drop of blood was placed in the centre the slide. The applicator stick was used to spread the drop in a circular pattern until the RBCs are completely haemolyzed and it has the size of a dime. The slides were laid on a smooth surface and allowed to air dry thoroughly at room temperature.

Thin film:

A small drop of blood was placed on a clean labelled slide near its frosted end. Another slide was placed at a 45-degree angle up to the drop, allowing the drop to spread along the contact line of the two slides and a thin layer of even spread was made. The thin smears were allowed to dry and later fixed by immersing them into absolute methanol for a minute.

Malaria thin and thick film microscopy.

The thick and thin blood films were stained using 10% Giemsa for 15 minutes then rinsed and allowed to air dry. Slides were examined under the microscope using the oil immersion objective lens. A slide was considered positive when at least one trophozoite of the *P. falciparum* parasite was identified. Parasitaemia was determined by counting the number of parasites per 200 white blood cells and assuming that each subject had 8000

white blood cells/µL of blood [19, 20]. The thin films were used to identify the Plasmodium specie present.

Procedure for RDT Test

Approximately 5µL of blood were used to diagnose malaria using the malaria antigen *Plasmodium falciparum* RDT kit, following the manufacturer's instructions. The *P. falciparum* antigen rapid diagnostic test (RDT) kit is a diagnostic tool used to detect the presence of *P. falciparum* antigens in a patient's blood. This test is based on the detection of specific proteins produced by the malaria parasite, which are released into the bloodstream during an infection. When a small amount of blood was applied to the sample pad on the test strip, it flowed by capillary action through the strip and reached the area where the antigens are bound to the antibodies. Two drops of buffer were added to the cassette of the RDT and left for 10 minutes to migrate after which the results were read. If the *Plasmodium falciparum* antigens were present in the blood sample, they bound to the antibodies on the test strip, forming a visible coloured line.

Data management and analysis

Questionnaires from participants were properly checked for completion and stored in a safe cupboard at the end of the day after data has been collected. Samples were placed in a safe box and transported to the health facility for analysis. Data obtained from questionnaires and laboratory tests were entered into Microsoft Excel. A copy of the excel file generated at the end of data collection was exported to SPSS version 25.0 for cleaning and analysis. The questionnaires were kept in a locked cupboard in case needed for reference.

Parasite density (Parasite/µL of blood) was calculated using the WHO recommended assumed WBC count of 8000µL of blood:

Parasite density= (number of asexual parasite counted)/ (WBC counted) X 8000/µL blood

Prevalence of malaria was calculated by dividing the number of positive samples by the total sample size for mRDT and microscopy:

Malaria Prevalence= (number of asexual positive samples)/ (Total number of participants) X 100

Descriptive statistics was used to summarize the demographic and clinical characteristics of the participants.

The sensitivity, specificity, positive predictive value (PPV) and negative predictive value (NPV) of the SD Bioline malaria antigen RDT was calculated using standard formulas and obtained along with 95% CI (confidence interval).

The diagnostic accuracy of the RDT was assessed using a standard formula and confirmed by the receiver operating characteristic (ROC) curve. The area under the ROC curve (AUC) was analysed to determine the overall accuracy of the RDT (AUC). For the AUC, values range from 0.5 to 1.0 where 0.5 was considered to be indicative of chance performance and 1.0 was considered perfect performance (higher values indicate better performance). Logistic regression analysis was performed to determine any demographic or clinical factors that may affect the results of the RDT. Chi-square test was used to determine association between parasite density and RDT positivity. The degree of agreement between the malaria RDT and microscopy was scaled following the Cohen's Kappa analysis; 0.0-0.2 was considered poor, 0.2-0.4 was considered fair, 0.4-0.6 was considered average, 0.6-0.8 was considered good and 0.8-1.0 was considered excellent. Statistical significance was set at a p-value of <0.05.

Sensitivity= (True positive)/ (True Positive + False Negative) X 100

Specificity= (False positive)/ (False negative + True positive) X 100

Positive Predictive Value (PPV) = (True positive)/ (True positive False Negative) X 100

Negative Predictive Value (NPV) = (False Positive)/ (False Negative + True Positive) X 100

Accuracy= (True Positive + False Negative)/ (True Positive + False Positive + False Negative + True Negative) X 100

Were confirmed by two-by-two contingency table.

RESULTS

Sociodemographic characteristic of study participants

A total of 250 participants participated in the study and were screened for malaria parasites (Table 2). All of the participants were recruited from the Tombel Health Area. Their age ranged from 2-70 years, with 108(43.2%) of the participants being within the age group 31-45 years with a mean age (±SD) of 31. 1 (±21.5) and median age of 27. Among study participants, 160 (64%) were females and 90 (36%) were males. Also, about 97 (38.8%) of the participants were reported to be Married/Cohabitating and 141 (56.4%) were seen single while 12 of them were widowed (4.8%). Majority of the participants were students 69(27.6%) followed by Business people 57 (22.8%) and the least of them were health personnel 4 (1.6%). Primary level of education was the most frequent level 105 (42.0%) attained by the participants followed by secondary level 72 (28.8%) and least was the tertiary level 14 (5.6%). Twenty-eight (11.2%) of the participants had no formal education. Christians dominated the study participants 221 (84.0%) and Muslims were 29 (11.6) of the total study population. Table 2 shows a summary of the socio-demographic characteristics.

Table 2: Sociodemographic characteristic of study participants

Variables	Category	No (%)
**Sex**	Male	90(36.0)
	Female	160 (64.0)
	**Total**	**250 (100)**
**Age group (Years)**	<5	26 (10.4)
	5-15	13 (5.2)
	16-30	38 (15.2)
	31-45	108 (43.2)
	46-60	34 (13.6)
	60+	31 (12.4)
	**Total**	**250 (100)**
**Occupation**	Business	57 (22.8)
	Farmer	48 (19.2)
	Health personnel	4 (1.6)
	House wife	15 (6.0)
	None	52 (20.8)
	Student	69 (27.6)
	Teacher	5 (2.0)
	**Total**	**250 (100)**
**Marital Status**	Single	141 (56.4)
	Married	97 (38.8)
	Widowed	12 (4.8)
	**Total**	**250 (100)**
**Highest Level**	Primary	105 (42.0)

	Secondary	72 (28.8)
	High school	31 (12.4)
	Tertiary	14 (5.6)
	None	28 (11.2)
	**Total**	**250 (100)**
**Religion**	Christian	221 (88.4)
	Muslim	29 (11.6)
	**Total**	**250 (100)**

Prevalence of malaria in the Tombel district area

Majority of the participants-143 (57.2%) presented with symptoms for malaria. Sixty participants (24% [95% CI:19 - 29]) out of 250 were found positive for the malaria RDT (Figure 1) while 133 (53.2% [95% CI :47% - 59]) were positive with microscopy (Figure 1). There is a statistically significant difference between the prevalence of malaria using RDT and its prevalence using microscopy with a P-value <0.001. The most prevalent *Plasmodium specie* was *P. falciparium* found in 133 samples (53.2%). Of the 133 samples that had *P. falciparium,* 3 of the samples had mixed infection of *P. falciparium* and *P. malariae* (Table 3).

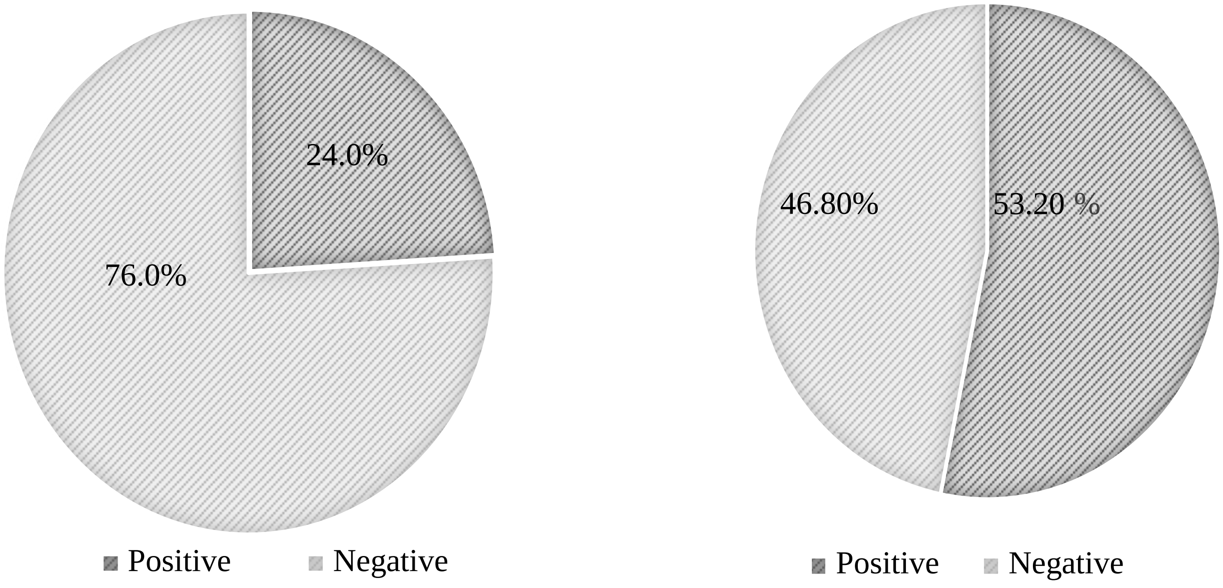

P-value <0.0001

Figure 1: Prevalence of malaria using SD Bioline Ag *P. falciparum* test (left) and Microscopy (right)

Table 3: Prevalence of malaria symptoms and malaria with malaria RDT and microscopy

Variable	Category	No (%)	95%CI
**Presence of symptoms**	NO	107 (42.8)	32-51.7
	YES	143 (57.2)	51.1-63.3
	**Total**	**250 (100)**	
**RDT**	Negative	190 (76)	70.7-81.3
	Positive	60 (24)	18.7-29.3
	**Total**	**250 (100)**	
**Microscopy**	Negative	117 (46.8)	40.6-53.0

	Positive	133 (53.2)	47.0-59.4
	**Total**	**250 (100)**	
**Specie type**	None	117 (46.8)	40.6-53.0
	P. falciparium	130 (52.0)	45.8-58.2
	P. malariae and *P. falciparium*	3 (1.2)	-0.1-2.5
	**Total**	**250**	**100**

Validity of SD BIOLINE to diagnose *P. falciparum* and concurrence with Microscopy

A total of 250 samples were tested both for Microscopy and RDT. One hundred and thirty-three (133) samples were found positives with microscopy, seventy-three samples among the positives with microscopy turned out to negative with RDT (FN = 73). Sixty (60) samples were then Positive with RDT (TP = 60) and all of the 60 positives samples that were positives with RDT, were also positive with microscopy (FP = 0). Out of the one hundred and ninety (190) samples that were tested negative with RDT, one hundred and seventeen (117) were also negative with microscopy. The mRDT correctly identified 60 (TP) malaria cases out of 133 that were identified with microscopy and missed 73 (FN) malaria cases (Table 4).

Table 4: Validity of SD BIOLINE to diagnose *P. falciparum* and concurrence with Microscopy

		**Microscopy**		**Total**
		**Positive**	**Negative**	
**mRDT**	**Positive**	60	0	60
	**Negative**	73	117	190
	**Total**	133	117	250

The sensitivity and specificity were 45% (95% CI:37–54) and 100% (95%CI:96–100), respectively while the Positive Predictive Value (PPV), Negative Predictive Value (NPV) and accuracy were 100% (95% CI:94–100), 61.50% (95% CI:54–68) and 70.8% (95% CI:65–76) respectively for the malaria RDT (Table 5). Cohen's Kappa analysis revealed a moderate degree of agreement between mRDT and microscopy (K = 0.435, $p<0.001$) (Table 5).

Table 5: Performance of mRDT in diagnosis of *P. falciparum* compared to reference standard and studies in Cameroon

Measure	RDT% (95 %CI)
**Sensitivity**	45 (37-54)
**Specificity**	100(96-100)
**PPV**	100(94-100)
**NPV**	61.50(54-68)
**Kappa value**	0.435(0.369 - 0.435)
**Accuracy**	70.8(65-76)
**AUC value**	0.78

Association between malaria parasites density and RDT positivity test

There was a statistically significant positive and moderate correlation between parasite density for malaria and malaria RDT ($r = 0.459$, $p < 0.001$). (Table 6, 7).

Table 6: Correlation between Malaria parasites density and RDT positivity

	Parasite density	RDT

**Microscopy (Trophozoites/mm3)**	Pearson Correlation	1	0.459
	P-value		0.000
	N	250	250
**RDT**	Pearson Correlation	0.459	1
	P-value	0.000	
	N	250	250

Table 7: Association between Malaria parasites density and RDT positivity

**Parasite density**	**RDT**		**Chi-square**	**P-value**
	**Positive (%)**	**Total (%)**		
**Low**	52 (20.8)	121 (48.4)	72.809	<0.001
**Intermediate**	8 (3.2)	12 (4.8)		
**High**	0 (0.0)	117 (46.8)		
**Total**	60 (24)<br>24.0%	250 (100)<br>100.0%		

DISCUSSION

In order to control malaria, a high-quality diagnostic technique must be used to identify the parasite before anti-malarial medication is prescribed in accordance with WHO guidelines. Diagnosing malaria parasites allows for early parasite identification, supports characterization of the treatment response, and focuses treatment efforts [21].

The prevalence of malaria with the malaria RDT was found to be 24% with the SD BIOLINE Malaria Ag. RDT and 53.2% with microscopy in Tombel area with statistically significant difference ($p < 0.05$). This disparity could be attributed to some of the inherent and external factors, like the stability of the RDT which include exposure to extreme temperatures, which are a major contributor to poor performance of RDT, especially during transport as well as storage as observed by previous research. The prevalence with mRDT is lower than that recorded by Bamidele et al in Nigeria who had 53% [22] and Samadoulougou and collaborators in Burkina Faso who had 65% [23]. Also, in a study conducted in the Far North region of Cameroon, the overall malaria prevalence of 34.9% using mRDT and 59.2% using microscopy was reported by Tchinda et al [24]. In a study in the North West region of Cameroon, Kimbi and collaborators reported a malaria prevalence of 26.5% using mRDT which is similar to our study and 37.5% using microscopy which lower than recorded in our study [25]. These differences in prevalence may be due to the difference in endemicity of malaria from the different malaria epidemiological zones where the studies were carried.

Malaria prevalence was higher in females than males by microscopy and the findings are in line with Kimbi and collaborators [26] who reported that females spend more time outdoors at dusk and dawn than males to perform household chores and as such are more exposed to mosquito bites. Those aged 31-45 had a higher prevalence compared to the other age ranges which is contrary to the findings of Ning et al where children <5 years of age were more infected [26]. This could be explained by the fact that since it was a rural

setting, the older adults are those involved with farming activities and businesses that keeps them outdoors exposes them to mosquito bites.

There was a statistically significant strong positive association between the presence of malaria symptoms and the mRDT positivity of (P= 0.023). In other words, individuals who reported malaria symptoms were more likely to test positive for malaria compared to those who did not have symptoms (OR = 2.056 times [95%CI; 1.10-3.82]). This is similar to a study carried out by Deus and collaborators 2019 in Tanzania who reported a statistically significant strong positive association between presence of malaria symptoms and RDT positivity.

The species of malaria parasite identified in all cases were *P. falciparum* (97.8%) and *P. malariae* (2.2%) that was identified in three cases. These results are similar to a study carried out by Oyeyemi and collaborators [27] in South-Western part of Nigeria comparing microscopy and rapid diagnostic test as malaria diagnostic tools where *P. falciparum* species of the malaria parasites was identified in all the cases. Also, Agomo and collaborators [22] in Lagos, Nigeria, found that *P. falciparum* was seen in 95.2% of the cases as mixed infection with *P. malariae* (3.6%). These results agree with literature findings that *P. falciparum* is responsible for more than 95% of malaria infections in the tropics.

The sensitivity and specificity of the RDT were 45% and 100% respectively. A sensitivity of 45% means that the test correctly identified 60 (45%) of true positive *P. falciparium* cases while specificity of 100% implies that the test correctly identified all negative cases. Also, the high PPV of 100% suggests that if the mRDT identifies a participant as positive for malaria, there is a very high likelihood that they are truly infected. However, the lower NPV of 61.5 % suggests that if the test identifies a person as negative for malaria, there is still a chance that they might be infected. The lower

sensitivity may due the quality of the RDT used and the prevalence of malaria in our study population. The findings of our study contradict the findings of Kimbi and collaborators [25] who reported a sensitivity and specificity of 82.4% and 76.6% respectively. Their study also reported a PPV of 57.4% and NPV of 91.9% which is in contrast with our findings. Another study conducted in the South West region of Cameroon by Wanji and collaborators [26] reported findings similar to our study with a specificity being 98.3% and slightly different PPV of 95.9% but higher sensitivity of 91.2% and NPV of 97.3%. These differences may be due to the difference in study population and difference in prevalence of the disease. A study by Maltha and collaborators [27] in Burkina Faso demonstrated higher sensitivity, specificity, PPV and NPV of 100%, 70.9%, 69.4% and 100%, respectively, for PfHRP2 detection. Likewise, a study by Diongue *et al*. [28] in Senegal reported that Carestat RDT showed high sensitivity (97.3%) and specificity (94.1%) with PPV and NPV of 97.3 and 94.1% respectively. Several factors are reported to affect the sensitivity of RDTs based on detection of HRP-II, including an inherent limitation of the device, mutation or deletion of the gene encoding the HRP-II, and storage conditions. It is known that the sensitivity of the RDT is affected by low parasitic densities, and that below 100 parasites/µL the RDT performance decrease.

The area under the curve (AUC) was 0.78 for the receiver operating characteristic (ROC) curve of the malaria rapid diagnostic test (RDT) and this value suggests that the test has moderate diagnostic accuracy. The AUC of 0.78 means that the test has a 78% chance of correctly distinguishing between individuals who have malaria and those who do not have malaria. However, it also means that there is a 22% chance that the test will produce a false positive or false negative result. This level of accuracy may be acceptable in some contexts, but it is not ideal for a diagnostic test, particularly for a disease like malaria that can have serious health consequences if left untreated. The findings of this

study are similar to a study carried out by Teh and collaborators [29] in the Mount Cameroon area who reported an AUC of 0.75. This could be due to the similarity in the study population and prevalence of the disease. On the other hand, our finding is lower than that of a study carried out by Njunkeng and collaborators [30] in Cameroon who reported an AUC of 0.97. This disparity might be due to the difference in population type and prevalence. Another study carried out by Mbulli and collaborators [31] in Cameroon demonstrated a higher AUC of 0.90. This level of accuracy which is higher than that of our study may be associated to the fact that their study population had a high prevalence of malaria and their population were children <5 years who are 2 times more likely to be positive for mRDT than those older.

A significant association (P<0.001) was identified between the SD BIOLINE malaria Ag *P. f* RDT and the parasite density. This is similar to a study carried out by Teh and collaborators [29] in Cameroon which had a sensitivity of 96.1% at parasite density greater than or equal to 200 parasites/µL of blood, as recommended by WHO [32]. This is similar to the findings of Oyibo et al [33] in a study who identified a significant association between parasite density and RDT positivity in children aged 0-2 years in Nigeria, with higher parasite densities associated with greater likelihood of RDT positivity. Lopes et al [34] reported a similar finding for which there was a significant association between parasite density and RDT positivity for the SD Bioline Malaria Ag *P.f*. Another study carried out by Djoufounna et al. [35] found a significant association between parasite density and RDT positivity for the CareStart Malaria HRP2 RDT. This implies an increasing parasite density increased the sensitivity of the mRDT to detect the presence of the malaria parasite.

The test utilized for this investigation was based on the HRP2, which is the most widely used test for parasitological confirmation of malaria before treatment in sub-

Saharan Africa. Numerous studies have reported marked drops in HRP2 RDT sensitivity following reductions in P.f HRP2 deletion or transmission intensity. Parasites missing HRP2 are a potential source of false-negative HRP2 RDTs. Although RDTs are employed as diagnostic tools, diagnosis by microscopy should never be abandoned because it is the gold standard in endemic areas. In addition, microscopy allows the computation of parasite densities and identification of all species and is cheaper than the other approaches. PCR is still expensive even though it is the most effective diagnostic technique with excellent sensitivity and specificity.

CONCLUSION

The overall prevalence of malaria is high in the Tombel Health Area both with use of the mRDT and microscopy. SD Bioline Malaria Ag P.f used reported a high level of specificity and positive predictive value with relatively low sensitivity and negative predictive value. In addition, the overall accuracy of SD BIOLINE Malaria Antigen P.f HRP2 with microscopy as reference standard in population screening for *P. falciparum* infection in the community is moderate. The RDT has proven to be moderately effective as a diagnostic test for confirmation of clinical cases of malaria. This study shows a variation in the performance of the RDT with respect to the age. Hence this sociodemographic characteristic must be taken into account, in order to explain the results obtained. This, in a certain way, indicates that the use of RDTs is limited, it may be useful for diagnoses in remote areas, but for prevalence studies it is better to continue using microscopy as a reference technique or if it is possible the PCR. Finally, there is a significant association between parasite density and the RDT positivity where an increase

parasite density increases the sensitivity of the RDT (*P*<0.001). Microscopy remains a preferable option, when parasite density needs to be determined in the absence of PCR.

Author contributions DSN conceived the study; ALN carried out Methodology investigation; WD, ALN carried out data analysis. WD, ALN prepared the original draft; DSN, ODK, EMZ edited and reviewed the draft; The general supervision was carried out by DSN, WD; All authors read and approved the final version.

Funding: This research received no specific grant from any funding agency in the public, commercial, or not-for-profit sectors.

Data Availability The data generated or analysed during the current study are available from the corresponding author on reasonable request.

Conflict of interests: All authors declare no conflict of interests relevant to the content of this manuscript.

REFERENCES

1. WHO, World malaria report, The World Health Organization, Geneva, 2020.
2. Massoda Tonye SG, Kouambeng C, Wounang R, Vounatsou P. Challenges of DHS and MIS to capture the entire pattern of malaria parasite risk and intervention effects in countries with different ecological zones: the case of Cameroon. Malaria Journal 2018;17(1):156.
3. MSP, Bulletin Epidémiologique annuel 2018 du Paludisme au Cameroun, Ministry of Public Health, Cameroon. 2018; 364 :1896-8

4. Minsante. XIième Journée mondiale de lutte contre le paludisme “prêt à vaincre le paludisme” Nous sommes la génération qui peut éliminer le paludisme. Dossier de Presse, Minsante. 2018

5. WHO. World malaria report 2015: World Health Organization; Geneva 2015.

6. Looareesuwan S. Malaria. In: Looareesuwan S, Wilairatana P, editors. Clinical Tropical Medicine. 1st ed. Bangkok, Thailand: Medical Media 1999; 364:1896-8

7. Amexo M, Tolhurst R, Barnish G, Bates I. Malaria misdiagnosis: Effects on the poor and vulnerable. Lancet 2004; 364:1896-8.

8. Makler MT, Palmer CJ, Ager AL. A review of practical techniques for the diagnosis of malaria. Annals of Tropical Medicine and Parasitology 1998; 92:419-33.

9. UNICEF. Malaria diagnosis: A guide for selecting rapid diagnostic test (RDT) kits. 1st Moody A. Rapid diagnostic tests for malaria parasites. Clinical Microbiology Revolution 2002; 15:66-78

10. Jorgensen P, Chanthap L, Rebueno A, Tsuyuoka R, Bell D. Malaria rapid diagnostic tests in tropical climates: The need for a cool chain. American Journal of Tropical Medicine and Hygiene 2006; 74:750-4.

11. Albertini A, Lee E, Coulibaly SO, Sleshi M, Faye B, Mationg ML, et al. Malaria rapid diagnostic test transport and storage conditions in Burkina Faso, Senegal, Ethiopia and the Philippines. Malaria Journal 2012; 364:1896-8

12. Shillcutt S, Morel C, Goodman C, Coleman P, Bell D, Whitty CJ, et al. Cost-effectiveness of malaria diagnostic methods in sub-Saharan Africa in an era of combination therapy. Bull World Health Organ 2008; 86:101-10.

13. Elechi HA, Rabasa AI, Bashir MF, Gofama MM, Ibrahim HA, Askira UM. Uncomplicated malaria in children: The place of rapid diagnostic test. Nigeria Medical Journal 2015; 56:85-90.

14. Melle EM, Etane SM, E LM. Primate bush meat consumption: A source of zoonotic Disease Risk in Tombel Area, South-West Region, Cameroon. International journal of environmental Sciences and Natural Resources 2015; (2) 39-46.

15. Sharlot F, Amelie DM, Doroty FA, Rinelle ND, Andre LA, Alain MD. The impact of climate change on malaria incidence in Bonaberi and Tombel, Cameroon. Research Square. 2023; Short Report DOI: https://doi.org/10.21203/rs.3.rs-3258929/v1.

16. Buderer NMF. Statistical methodology: I. Incorporating the prevalence of the disease into the sample calculation for sensitivity and specificity. Academic Emergency Medicine 1996; 3(9): 895-900 DOI: https://wnarifin.github.io/ssc/ssmcnemar.html

17. Nyasa RB, Fotabe EL, Ndip RN Trends in malaria prevalence and risk factors associated with the disease in Nkongho-mbeng; a typical rural setting in the equatorial rainforest of the South West Region of Cameroon. PLoS ONE 2021; 16(5): 251-380.

18. Kouitcheu MLB, Noundjeu NML, Leundji H. Potential risk factors and prevalence of *Helicobacter pylori* infection among adult patients with dyspepsia symptoms in Cameroon. BMC Infectious Diseases 2018 9;18(1):1-1.

19. Ndamukong-Nyanga JL, Kimbi HK. Sumbele IUN, Nana Y, Bertek SC, Ndamukong KJN, Lehman LG. A Cross- Sectional study on the influence of

Altitude and urbanization on the coinfection of malaria and Soil-transmitted helminthes in Fako Divisionm, south West Cameroon. International Journal of Tropical Diseases and Health. 2015;8(4): 150-164.

20. Nyanga JLN, Patakinzo CG, Ngum CN, Desdemona NN and Nyanga BY. The Influence of Helicobacter pylori Infection on Malaria Parasitaemia among Symptomatic Patients in Buea, Cameroon. International Journal of Tropical Disease and Health 2018; 31(4): 1-7,

21. Falade CO, Ajayi IO, Nsungwa-Sabiiti J, Siribié M, Diarra A, Sermé L, et al. Malaria rapid diagnostic tests and malaria microscopy for guiding malaria treatment of uncomplicated fevers in Nigeria and pre-referral cases in 3 African Countries. Clinical Infectious Diseases 2016;63-65.

22. Ogunfowokan O, Ogunfowokan BA, Nwajei AI. Sensitivity and specificity of malaria rapid diagnostic test (mRDT CareStatTM) compared with microscopy amongst under five children attending a primary care clinic in southern Nigeria. African Journal of Primary Health Care and Family Medicine 2020;12(1), a2212

23. Samadoulougou S, Kirakoya-Samadoulougou F, Sarrassat S et al. Paracheck® rapid diagnostic test for detecting malaria infection in under five children: A population- based survey in Burkina Faso. Malaria Journal 2014; 13:101-103.

24. Tchinda VH, Tchinda DM, Mbacham WF. Malaria in Cameroon: burden and strategies for control and elimination. Journal of Public Health in Africa 2019; 10(1): 1057-1060.

25. Kimbi HK, Nkesa SB, Ndamukong-Nyanga JL, Sumbele IUN, Atashili J, Atanga, M B. Knowledge and perceptions towards malaria prevention among vulnerable

groups in the Buea Health District, Cameroon. BMC Public Health 2018; 18(1): 1084-1090.

26. Wanji S, Kimbi HK, Eyong JE, Tendongfor N and Ndamukong JL. Performance and usefulness of the Hexagon rapid diagnostic test in children with asymptomatic malaria living in the Mount Cameroon region. Malaria Journal 2008 ; 7:89 doi:10.1186/1475-2875-7-89

27. Maltha J, Guiraud I, Lompo P, Kaboré B, Gillet P, Van Geet C, et al. Accuracy of Pf HRP2 versus Pf-pLDH antigen detection by malaria rapid diagnostic tests in hospitalized children in a seasonal hyperendemic malaria transmission area in Burkina Faso. Malaria Journal 2014; 13:20

28. Diongue K, Ndiaye M, Gaye A, Deme A, Badiane AS, Ndiaye D. Evaluation of CareStart ™ Malaria HRP2/pLDH (Pf/pan) Combo Test in a malaria low transmission region of Senegal. Malaria Journal 2017; 16:328.

29. Teh RN, Sumbele IUN, Asoba Nkeudem, G, et al. Concurrence of CareStart™ Malaria HRP2 RDT with microscopy in population screening for *Plasmodium falciparum* infection in the Mount Cameroon area: predictors for RDT positivity. Tropical Medicine and Health 2019; 17: 47-60.

30. Njukeng, Paul A. Asumke N. "Evaluation of the diagnostic performance of CareStart™ malaria HRP2/pLDH (Pf/pan) combo test for malaria diagnosis in a clinical setting in Cameroon". Malaria Journal 2019;16(1):372-378.

31. Mbulli IA, Akindeh MJ, Bonkum JC, et al. Diagnostic accuracy of CareStart™ Malaria HRP2 and SD Bioline Pf/PAN for Malaria in febrile outpatients in varying Malaria Transmission settings in Cameroon. Diagnostics 2021; 11(9): 1556.

32. Bell D, Cunningham J. Malaria rapid diagnostic test performance; results of WHO product testing of malaria RDTs: round 3 (2010–2011). Geneva: World Health Organization; 2011.

33. Oyibo WA, Folarin OA, Brieger WR, Salako LA, Saliu OA. Correlation of *Plasmodium falciparum* malaria parasite density measured by microscopy and rapid diagnostic test with incidence of fever in children aged 0-2 years in Nigeria. Malaria Journal 2013; 7:223-31

34. Lopes SCP, Oliveira LCG, Fontes CJF, Daniel-Ribeiro CT. Performance of three malaria rapid diagnostic tests in a non-endemic area of Brazil: The importance of *Plasmodium falciparum* malaria. International Journal of Infectious Diseases 2019; 171-177.

35. Djoufounna J, Mayi MPA, Bamou R, Foyet JV, Tabue R, Lontsi-Demano M, Achu-Fosah D, Antonio-Nkondjio C, Tchuinkam T. High prevalence of asymptomatic *Plasmodium falciparum* malaria in Makenene, a locality in the forest-savannah transition zone, Centre Region of Cameroon. Current Research in Parasitology & Vector-Borne Diseases 2022; 2: 100104

Click here to access/download
Supporting Information
Ngalameh Ethical clearance.pdf

Click here to access/download
Supporting Information
Ngalameh Consent form.pdf

Click here to access/download
Supporting Information
NGALAME ET AL MANUSCRIPT trash.docx

Click here to access/download
Supporting Information
Ngalame et al Manuscript revised. Trash 26 march doc.doc
