# Supplementary material for: Assessment of the diagnostic performance of the SD Bioline Malaria antigen test for the diagnosis of malaria in the Tombel health district, Southwest region of Cameroon
Source: PLoS One. 2025 Mar 13;20(3):e0298992. doi: 10.1371/journal.pone.0298992 (PMC11906078; doi:10.1371/journal.pone.0298992)
Supplement: Attachment — Submitted filename: review malaria _020945.doc [file pone.0298992.s007.doc]

# Research Paper author Review

## General comment

Overall, the research has a good objective, sensitivity, specificity, positive predictive value and negative predictive value (the study aims to evaluate the diagnostic performance of the particular RDT in one particular setting). The study was conducted with full ethical standards and received ethical clearance, as well as administrative authorizations and an informed consent from participants. The weaknesses of the study include a limited range because the research was focused only on the diagnostic performance of the one type RDT in the one particular region, so can hamper its generalization in other settings. Noted sample size , while the sample calculation was included in the methodology chapter, the final 250 individuals might be insufficient for the appropriate approximation.

## Abstract

This article provides a thorough evaluation of the Ag P.f RDT's diagnostic accuracy for detecting malaria in the Tombel Health Area. While the RDT showed high specificity and positive predictive value, its sensitivity was lower, suggesting possible missed cases of malaria. The research highlights the need for more effective diagnostic approaches in resource-constrained environments.

### Introduction

The introduction effectively places the study in the context of the global malaria burden, stressing the importance of precise diagnosis. However, it does not acknowledge the documented cases of artemisinin antimalarial drug resistance or address the challenges associated with RDT tests. Nevertheless, the study's", emphasis on assessing the diagnostic value of the SD BIOLINE malaria antigen test addresses an important research gap.

## Literature Review

Although previous studies are briefly mentioned in the introduction, a deeper review of RDT performance literature in similar contexts could enhance the study's foundation and its alignment with existing research.

##  Method and Material

More information on statistical methods for data analysis is needed in the manuscript. It is suggested to broaden the study's scope by including various RDT types and increasing the sample size for greater reliability.

## **Discussion**

In the discussion section, the factors affecting RDT performance are examined, but a more thorough analysis and recommendations for improvement would enhance practical relevance for healthcare professionals and policymakers. The study's results reveal the prevalence of malaria in the Tombel Health Area and emphasize the importance of considering sociodemographic factors when interpreting RDT outcomes. While RDTs are useful, microscopy or PCR tests are still favored for prevalence studies. The discussion points out differences between RDT and microscopy findings, stressing the need to address these inconsistencies in future research endeavors.

## Conclusion

In conclusion, microscopy remains the preferred option for malaria diagnosisThe discussion section emphasizes the importance of accurate malaria diagnosis for effective treatment, aligning with WHO guidelines. It highlights the prevalence disparities between RDT and microscopy results, possibly influenced by factors like RDT stability and endemicity variation across regions. Gender and age differences in prevalence are noted, attributed to outdoor exposure and occupation patterns.
