# Supplementary material for: Assessment of the diagnostic performance of the SD Bioline Malaria antigen test for the diagnosis of malaria in the Tombel health district, Southwest region of Cameroon
Source: PLoS One. 2025 Mar 13;20(3):e0298992. doi: 10.1371/journal.pone.0298992 (PMC11906078; doi:10.1371/journal.pone.0298992)
Supplement: Attachment — Submitted filename: Ngalame et al RESPONSE TO REVIEWERS.doc [file pone.0298992.s011.doc]

PONE-D-23-43663

Response to Reviewers

June 16^th^, 2024

Dear Dr Enoch,

Thank you for giving us the opportunity to submit a revised draft of the manuscript entitled “*Assessment of the diagnostic performance of the SD Bioline malaria antigen Plasmodium falciparum rapid diagnostic test for the diagnosis of malaria in the Tombel health area, Southwest region of Cameroon*” for publication in the *PLOS ONE*. We appreciate the time and effort that you and the reviewers dedicated to providing feedback on our manuscript and are grateful for the insightful comments on and valuable improvements to our paper. We have incorporated most of the suggestions made by the reviewers. Those changes are highlighted within the manuscript. Please see below, in blue, for a point-by-point response to the reviewers’ comments and concerns.

**Reviewers' Comments to the Authors:**

**Reviewer 1**

a. Comment from Reviewer 1: The sample size calculation is unclear. The authors mention using the Burderer method but do not provide sufficient details on the assumptions and values used in the calculation.

**Authors’ response:** Thank you for pointing this out. The reviewer is correct,

We used the sample size calculation method of Burder and collaborators 1996 as follow:

n = n_1_ + n_2_

And

Where,

n1 and n2 = minimum required number of participants for sensitivity and specificity with estimated sensitivity and specificity of 95% and 99% respectively

TP = True Positive (Positive for malaria with microscopy)

TN = True Negative (Negative for malaria with microscopy)

FN = False Negative (Positive for malaria with microscopy but negative with the RDT)

FP = False Positive (Negative for malaria with microscopy but positive with the RDT)

P = prevalence of malaria from previous study (34.7% gotten from a study carried out by Ngum and collaborators., 2023 in Mezam division, North-West Region, Cameroon) [73].

Z = standard normal variation at a confidence level of 95% (standard value of 1.96)

W = 0.05 (5% maximum acceptable width of the 95% confidence interval)

Substituting in the equation above,

TP+FN=〖1.96〗^2 (0.95(1-0.95))/〖0.05〗^2

TP + FN = 72.99

TN+FP=〖1.96〗^2 (0.99(1-0.99))/〖0.05〗^2

TN + FP = 15.21

n1=72.99/0.347

n1	= 210.3

n2=15.21/ (1-0.347)

n2 = 23.3

n1 required for sensitivity = 211 participants n2 required for specificity = 24 participants (at least n = 235 participants).

Taking into account a 10 % non-response rate, our minimum sample size will be 258.5 ≈ 260 participants.

Due to the fact that we had a shorter period for data collection, we were unable to get more than 250 samples.

We have provided the detail in the manuscript.

b. Comment from Reviewer 1: The authors used a consecutive sampling technique based on the quarters in the Tombel Health Area. However, they do not provide a clear rationale for this approach or discuss potential limitations, such as selection bias.

**Authors’ response:** Thank you for pointing this out. This is true, but out of the 11 identified quarters, we randomly selected 9 which is representative of the population and we used probability proportionate to size to get the sample size per quarter. Updated in the manuscript

c. Comment from Reviewer 1: The authors do not mention blinding of the microscopists to the RDT results. This is important to avoid bias in the interpretation of the microscopy results.

**Authors’ response:** Thank you for pointing this out. That was not mentioned, was an error, but the microscopist was blinded of the RDT results, gotten in the field by the principal investigator and the thick and thin film slides were read in the Tombel health district by a trained microscopist.

Added in the manuscript

d. Comment from Reviewer 1: The data analysis section lacks detail. The authors should provide more information on how they handled missing data, if any, and how they calculated the 95% confidence intervals for the performance measures.

**Authors’ response:** Thank you for pointing this out. Data was keyed in by the principal investigator and this was done immediately after collection same day eliminating room for missing data. Updated in the manuscript

e. Comment from Reviewer 1: The authors should provide a more in-depth comparison of their findings with other studies and discuss potential reasons for the differences observed. They should also discuss the implications of their findings for malaria diagnosis and control in the study area and similar settings.

**Author response:** We agree with the reviewer’s assessment. Accordingly, throughout the manuscript, we have revised

f. Comment from Reviewer 1: The authors should provide more information on the training and experience of the microscopists who interpreted the blood films.

**Author response:** Thank you for pointing this out. The microscopist is a trained and professional microscopist working for the Tombel district Hospital. It has been added in the manuscript

g. Comment from Reviewer 1: The proofreading is mandatory

**Author response**: The entire manuscript has been proofread.

h. Comment from Reviewer 1: The authors should ensure that all references are formatted consistently and following the journal's guidelines.

**Author response:** Thank you for pointing this out. The reviewer is correct, and the references are updated

**Reviewer #2:**

The study does not appear to be sound and the flow of the manuscript was inconsistent and did not meet the PLOS ONE publication criteria. In particular, the order of the scientific flow was not maintained in the Methods and Materials section, and there was controversial information in the manuscript.

**Author response:** While we appreciate the reviewer’s feedback, we respectfully disagree. We think this study makes a valuable contribution to the field because the study provides valuable information on the accuracy of the RDT used in the THA and its potential usefulness in improving the diagnosis and treatment of malaria in rural areas for the PLOS ONE’s readers. The findings from this study will inform policy makers, healthcare providers, and other stakeholders involved in the management of malaria, and ultimately contribute to the reduction of the burden of this disease in rural communities.

1. Comment from Reviewer 2: The study period in the Abstract was “March to May 2023" and in the Materials and Methods section "February to July 2023".

**Author response:** Thank you for pointing this out. The reviewer is correct, that was a typographical error. Study period was from February to July 2023 comprising protocol development, collection of data, data management and analysis. The period of April to June was only for data collection, corrected in the manuscript

2. Comment from Reviewer 2: The total population of the study area was not clear with large differences; the author has given an approximate total (65,000 people) and the sum of the numbers of males, females and children 0-16 years (89,178 people). See page 13 Study area and setting section paragraph 1 “The THD is situated in a rural area, with a population of approximately 65000 people. The major sociological groups are Bakossi, Grass landers, Nigerians, Banyangi, Oroko. In general, the population consists of-Approximately 35,690 males Approximately 28,587 females– And about 24 901 children aged 0-16 years”.

**Author response**: Thank you for this suggestion. The population size was a representation of males and females **with** children reason we had approximately 65,000 thousand people (35,690 males + 28,587 females = 64227 which is approximately 65000). “With” replaced by “and” in the manuscript

3. Comment from Reviewer 2: The author stated in the Methods and Materials section that the study population was > 5 years and above, and in the Results section that the author included > 2 years of age. Also, the author does not explain in the inclusion and exclusion criteria why only age > 5 years and above and not under 5 years

**Author response:** Thank you for pointing this out. The reviewer is correct, it was a typographical error, participants were all age. Corrected in the manuscript

4. Comment from Reviewer 2: The sample size mentioned by the author in the abstract was 250, in the sample size determination 239, in the proportional allocation in table1 260, and in the result section 250, so which was the correct sample size??

**Author response:** Thank you for pointing this out. The details for this section have been provided in the manuscript and has been explained (see author’s response to comment a. from reviewer 1).

5. Comment from Reviewer 2: The author also used a consecutive sampling technique, why consecutive? Because it leads to bias or not representative to the population in the area, one of the reasons to use consecutive sampling technique was limited case or study population, but in this study the study was community based, so there was no challenge to get the determined sample size in the aforementioned study period. The way the sample was allocated was also not clear.

**Authors’ response:** This is true, but out of the 11 identified quarters, we randomly selected 9 which is representative of the population and we used probability proportionate to size to get the sample size per quarter.

Comment from Reviewer 2: The technical details were also unclear and had no scientific basis. For instance; data collection and laboratory diagnosis, the author does not follow the guidelines or SOPs of the given laboratory at all during the procedures and examination.

1. Who was the data collector?

**Authors’ response:** Thank you for pointing this out. Principal investigator who is also a certified and experienced laboratory scientist; Ngalame Abigail. Was added in the manuscript

2. Who carried out the laboratory investigation?

**Authors’ response:** Thank you for pointing this out. A trained microscopist employed at the Tombel District Hospital: Mr. Macleo. Was added in the manuscript

3. Comment from Reviewer 2: What was the scientific basis regarding to the way of thick and thin film preparation?

**Authors’ response:**

**Thick film;**

1.	The slides were labelled with the participant's unique identification number

2.	Approximately a drop of 6 µL of blood was placed in the centre the slide.

3.	The applicator stick was used to spread the drop in a circular pattern until the RBCs are completely hemolyzed and it has the size of a dime.

4.	The slides were laid on a smooth surface and allowed to air dry thoroughly at room temperature.

5.	Stained with giemsa, rinsed and let to air dry.

**Thin film;**

1.	Approximately a drop of 3 µL of blood was placed on a clean labelled slide near its frosted end.

2.	Another slide was placed at a 45-degree angle up to the drop, allowing the drop to spread along the contact line of the two slides and a thin layer of even spread was made.

3.	The thin smears were allowed to dry and later fixed by immersing them into absolute methanol for a minute.

4.	Stained with Giemsa, rinsed and let to air dry.

5.	Properly hemolysed RBCs retain the stain which was obvious after rinsing the stained slides

6. Comment from Reviewer 2: Data management and analysis was mixed with the laboratory examination; the parasite density calculation was to be included under microscopic examination but not the data analysis.

**Author response:** Thank you for pointing this out. Data management and analysis and the laboratory examination sections have been corrected

7. Comment from Reviewer 2: The statistical analysis was not carried out appropriately and rigorously; on page number ‘20” the author stated that the formula for calculating PPV and NPV was incorrect.

**Author response:** Thank you for pointing this out. The statistical analysis section has been updated

**Reviewer #3:**

Comment from Reviewer 3: The manuscript was conducted with full ethical standards and received ethical clearance, as well as administrative authorizations and an informed consent from participants. The weaknesses of the study include a limited range because the research was focused only on the diagnostic performance of the one type RDT in the one particular region, so can hamper its generalization in other settings. Noted sample size, while the sample calculation was included in the methodology chapter, the final 250 individuals might be insufficient for the appropriate approximation.

**Authors’ response:** Thank you for this suggestion. It would have been interesting to extend this study to other localities. However, this would not be possible because the focus was on the particular RDT constantly used in the Tombel District Hospital for testing malaria. Also, due to the fact that we had a shorter period for data collection, we were unable to get more than 250 samples. The justification has been added

**Reviewer #4:**

1- Comment from Reviewer 4: Please add some specificity in the writing "Malaria continues to be a serious global public health concern", I think it is a problem in certain regions of the world and not globally.

**Authors’ response:** As suggested by the reviewer, The revised text reads as follows *Malaria continues to be a serious global public health concern. The World Health Organization (WHO) reported	405,000 malaria deaths and 228 million cases	in 2018 worldwide in all endemic areas.*

2- Comment from Reviewer 4: please revise the English language

**Authors’ response:** Thank you for this remark. English language has been revised in general.

3- Comment from Reviewer 4: The sampling process is general and not specific how the samples were conducted "Study population

**Authors’ response:** reviewer 1 addressed the same issue in comment a. (has been answered)

Comment from Reviewer 4: All persons living in the Tombel Health Area aged from 5 years and above" I Think this is very general, please rewrite and specify

All persons living in the Tombel Health Area during recruitment of participants for the study aged from 2 years and above.

**Authors’ response:** reviewer 2 addressed the same issue in comment 3. (has been answered)

4- Comment from Reviewer 4: There is no statistical data or analysis to the data, please specify and mention the details

**Authors’ response:** reviewer 1 addressed the same issue in comment d. (updated in the manuscript)

5- Comment from Reviewer 4: There is no need to repeat the results data once as data in the text and in tables, please avoid redundancy

**Authors’ response:** Thank you for this remark. Text has been revised in general.

**Reviewer #5:**

**Authors’ response:** Thank you for all remarks. Those issues have been revised in the manuscript as indicated

- Comment from Reviewer 5: Title: too long correct it as “Assessment of the diagnostic performance of the SD Bioline malaria antigen test for the diagnosis of malaria in the Tombel health area, Southwest region of Cameroon”

- abstract: conclusion not clear

- Introduction: grammar problem

- Method: study design not clear, is it institutional / community based?

- Study Period: data collection period not clear, there is difference between the body of the manuscript and abstract.

- Have you done pretest for questionnaire?

**Reviewer #6:**

1. Abstract Section

- 22% chance that the test can produce a false result-this is there is high chance of false positive or false negative.

**Authors’ response:** The AUC of 0.78 means that the test has a 78% chance of correctly distinguishing between individuals who have malaria and those who do not have malaria. However, it also means that there is a 22% chance that the test will produce a false positive or false negative result.

- AUC…what it means?

**Authors’ response:** AUC = Area Under the (Receiver Operating curve; ROC) Curve

- Low sensitivity and Overall accuracy of P.f HRP2 in the THA is moderate so how you recommend for diagnosis of malaria.

**Authors’ response:** This level of accuracy may be acceptable in some contexts, but it is not ideal for a diagnostic screening test, particularly for a disease like malaria that can have serious health consequences if left untreated.

2. Materials and Methods

- Study Period -Different study period was noted (March to May 2023, February to July 2023,30th of April 2023 and was ended on the 20th of June 2023). Which is correct from this all?

**Author response:** Thank you for pointing this out. The reviewer is correct, that was a typographical error. Study period was from February to July 2023 comprising protocol development, collection of data, data management and analysis. The period of April to June was only for data collection, corrected in the manuscript
