# Supplementary material for: Assessment of the diagnostic performance of the SD Bioline Malaria antigen test for the diagnosis of malaria in the Tombel health district, Southwest region of Cameroon
Source: PLoS One. 2025 Mar 13;20(3):e0298992. doi: 10.1371/journal.pone.0298992 (PMC11906078; doi:10.1371/journal.pone.0298992)
Supplement: Attachment — Submitted filename: comment.docx [file pone.0298992.s009.docx]

**Comment**

- Title: too long correct it as “Assessment of the diagnostic performance of the SD Bioline malaria

antigen test for the diagnosis of malaria in the Tombel health area, Southwest region of Cameroon”

- abstract: conclusion not clear
- Introduction: grammar problem
- Method: study design not clear, is it institutional / community based?
- Study Period: data collection period not clear, there is difference between the body of the manuscript and abstract.
- Have you done pretest for questionnaire?
- In general the manuscript needs major revision
