# Supplementary material for: Assessment of the diagnostic performance of the SD Bioline Malaria antigen test for the diagnosis of malaria in the Tombel health district, Southwest region of Cameroon
Source: PLoS One. 2025 Mar 13;20(3):e0298992. doi: 10.1371/journal.pone.0298992 (PMC11906078; doi:10.1371/journal.pone.0298992)
Supplement: Attachment — Submitted filename: PONE-RESPONSE TO REVIEWERS-D-23-43663R2.docx [file pone.0298992.s012.docx]

Manuscript PONE-D-23-43663R2

Response to Reviewers

Dear Dr. Enoch,

Thank you for giving me the opportunity to submit a revised draft of my manuscript titled [Assessment of the diagnostic performance of the SD Bioline malaria antigen test for the diagnosis of malaria in the Tombel health area, Southwest region of Cameroon] to *[PLOS ONE]*. We appreciate the time and effort that you and the reviewers have dedicated to providing your valuable feedback on our manuscript. We are grateful to the reviewers for their insightful comments on our paper. We have been able to incorporate changes to reflect most of the suggestions provided by the reviewers. We have highlighted the changes within the manuscript.

Here is a point-by-point response to the reviewers’ comments and concerns.

**ABSTRACT**

**Comments from Reviewer 1**

1 **Comment 1:** [*The abstract should be rewritten in an unstructured format.]*

**Response:** Thank you for pointing this out. We agree with this comment. Therefore, we have rewritten the abstract in an unstructured format

2 **Comment 2:** [*Revise ‘The aim of this study was to assess the diagnostic performance Ag P.f RDT…’ to read ‘The aim of this study was to assess the diagnostic performance of the the SD Bioline Malaria Ag P.f RDT….]*

**Response:** Agree. We have, revised this point.

3 **Comment 3:** [*A consecutive sampling technique was used to collect blood samples of 250 individuals…were they asymptomatic individuals? Please clarify.]*

**Response:** Thank you for pointing this out, the samples were collected from symptomatic and asymptomatic individuals. This have been incorporated throughout the manuscript.

4 **Comment 4:** [*Microscopy identified 133 (53.2%) participants with malaria…clinical or asymptomatic?]*

**Response:** Thank you for pointing this out, the microscopy identified positive cases in both symptomatic and asymptomatic individuals. This have been incorporated throughout the manuscript.

5 **Comment 5:** [*Microscopy identified 133 (53.2%) participants include the parasite density range.]*

**Response:** Thank you for this suggestion. According to the results in table 7 we obtain express the parasites densities range for mRDT

6 **Comment 6:** [*Define mRDT on first mention.]*

**Response:** Agree. We have, revised this point.

7 **Comment 7:** [*mRDT identified 60 cases add % and include the parasite density range detected by the mRDT.]*

**Response:** Thank you for this suggestion. 52 (20.8%) were low parasites density and 8 (3.2%) were moderate parasites density and 0.00 for high density. It has been added in the manuscript.

**INTRODUCTION**

1 **Comment 1:** [*405,000 malaria deaths and 228 million cases in 2018.provide current data. How different is this statement from the succeeding one? Merge if possible.]*

**Response:** thank you for this suggestion. In April 2024, the new World Malaria report has mentioned that, there were 608 000 malaria deaths in 2022 in globally. African continent was home to 94% of malaria cases (233 million) and 95% (580 000) of malaria deaths and Over 90% of all malaria deaths occur in Sub-Saharan Africa. [3]. Has been corrected within the manuscript.

2 **Comment 2:** [*2.	Provide reference for ‘Compared to 625,000 in 2020, the expected number of malaria deaths in 2021 was 619 000. In Sub-Saharan Africa (SSA), the figures for cases and fatalities were 93% and 94% respectively’.]*

**Response:** thank you for this suggestion. This part has been replaced *with In April 2024, the new World Malaria report has mentioned that, there were 608 000 malaria deaths in 2022 in globally. African continent was home to 94% of malaria cases (233 milli on) and 95% (580 000) of malaria deaths and Over 90% of all malaria deaths occur in Sub-Saharan Africa.*

3 **Comment 3:** [*These include a lengthy return time; would you consider turnaround time?]*

**Response:** Thank you for pointing this out, we have, revised this point.

4 **Comment 4:** [*Authors use mRDT and in some places RDT, please be consistent.]*

**Response:** Thank you for pointing this out, we have, revised this point.

5 **Comment 5:** [*Define THA on first mention.]*

**Response:** Thank you for pointing this out, THA was replaced by Tombel Health District (THD) throughout the manuscript.

**METHODS**

6 **Comment 6:** [*Combine study design and study period to read ‘Study design and period’.]*

**Response:** Thank you for pointing this out, we have, revised this point.

6 **Comment 6:** [*Combine study design and study period to read ‘Study design and period’.]*

**Response:** Thank you for pointing this out, we have, revised this point.

7 **Comment 7:** [*In the abstract you mentioned that* *sample were collected from March to May 2023 but under the method sections, you mentioned 30th of April 2023 to 20th of June 2023. Please clarify these differences in the study periods.]*

**Response:** Thank you for pointing this out, sample were collected from 30th of April 2023 to 20th of June 2023. Has been corrected in the manuscript

8 **Comment 8:** [*What is the difference between THA and THD?]*

**Response:** Thank you for pointing this out, Tombel Health Area (*THA)* and Tombel Health District (*THD)* are the same area. THA has been replaced by THD throughtout the manuscript.

9 **Comment 9:** [*The Study area and setting section is too lengthy. Be brief by removing all irrelevant and unrelated information.]*

**Response:** Thank you for pointing this out, we have, revised this point.

10 **Comment 10:** [*Under inclusion/exclusion, write the information in continuous sentences.]*

**Response:** Thank you for pointing this out, we have, revised this point.

11 **Comment 11:** [*11.	Sample size determination. Your method looks quite confusing. Why don’t you use the cochrane formula once you know the prevalence of malaria from a previous study? If you are too strong about this method, show previous studies that used that method.]*

**Response:** Thank you for pointing this out, the sample size calculation proportions of sensitivity and specificity method of Burderer and collaborators 1996, cited in Educational Notes in Research Methodology and Medical Statistics by Fahim et al., 2019: DOI: 10.22114/ajem.v0i0.158. Has been added in the manuscript

12 **Comment 12:** [*Change Sampling technics to Sampling techniques.]*

**Response:** Thank you for pointing this out, we have, revised this point.

13 **Comment 13:** [*A drop of blood was placed in the centre the slide. State exact volume.]*

**Response:** Thank you for pointing this out. A drop of 6 µL of blood was placed at the centre the slide. Has been stated in the manuscript

14 **Comment 14:** [*Under thick film, please revise the information. It is not the applicator stick that hemolyses the samples and this is not why the sample is spread on the slide.]*

**Response:** Thank you for pointing this out, we have, revised this point.

15 **Comment 15:** [*Under thin film, state the exact volume as well.]*

**Response:** Thank you for pointing this out. Approximately 3 µL of blood was placed on a clean labelled slide near its frosted end. we have, revised this point in the manuscript.

16 **Comment 16:** [*Under microscopy, check the spelling of specie.]*

**Response:** Thank you for pointing this out, “The thin films were used to identify the ~~Plasmodium specie~~ *P. falciparum* present”. Has been corrected.

17 **Comment 17:** [*Procedure for RDT Test: Approximately 5μL of blood were used to diagnose malaria using the malaria antigen Plasmodium falciparum RDT kit, rephrase this statement. The volume of the blood is used to perform the test not used to diagnose malaria. Describe how the test was done and not the princples behind the test.]*

**Response:** Thank you for pointing this out. “Approximately 5μL of blood were used to ~~diagnose malaria~~ perform the test using the malaria Ag *P.f* RDT kit, following the manufacturer’s instructions. The mRDT cassette was label with participant identification and 5ul of blood was applied to the sample pad on the test strip. Immediately after the blood clotted, the applicator was firmly applied in the circle and two drops of buffer were applied vertically above the circle and left for 10 minutes to migrate after which the results were read. The results were read following the manufacturer’s instructions as: if a single pink color appears on the control line “C”, the result was considered as negative. If a pink colored band appears on the “C” control line and a distinct pink colored band also appears on Pf line, the result was considered as positive.

~~The Ag~~ *~~P.f~~* ~~RDT~~ ~~kit is a diagnostic tool used to detect the presence of~~ *~~P~~*~~.~~ *~~falciparum~~* ~~antigens in a patient's blood. This test is based on the detection of specific proteins produced by the malaria parasite, which are released into the bloodstream during an infection. When a small amount of blood was applied to the sample pad on the test strip, it flowed by capillary action through the strip and reached the area where the antigens are bound to the antibodies. If the~~ *~~P. falciparum~~* ~~antigens were present in the blood sample, they bound to the antibodies on the test strip, forming a visible coloured line.~~ The RDT results were obtained in the field by the principal investigator, a certified and experienced laboratory scientist”. Has been revised in the manuscript

**RESULTS**

1 **Comment 1:** [*Table 3: change NO to No. Same to YES.]*

**Response:** Thank you for pointing this out, we have, revised this point.

2 **Comment 2:** [*Define all the abbreviations under Validity of SD BIOLINE to diagnose P. falciparum and concurrence with Microscopy.]*

**Response:** Thank you for pointing this out, FN = False Negative; TP= True Positive; FP= False Positive we have revised in the manuscript.

3 **Comment 3:** [*Table of mRDT in diagnosis of P. falciparum compared to reference standard and studies in Cameroon. I did not see any previous study comparison.]*

**Response:** Thank you for pointing this out, **Table 5: Performance of mRDT in diagnosis of *P. falciparum* ~~compared to reference standard and studies in Cameroon~~**. Was a mistake, has been corrected

4 **Comment 4:** [*Table 7, how do you classify parasite count as low, high, moderate etc. Justify these classifications with reference to a published paper.]*

**Response:** Thank you for pointing this out, Parasite densities were graded using the ‘plus’ system scale: + (1 to 9 trophozoites in 100 fields); ++ (1 to 10 trophozoites in 10 fields); +++ (1 to 10 trophozoites per field); ++++ (>10 trophozoites per field) recommended by WHO. These scores were used to estimate parasite densities reported by Kosack et al [20] as low density = 10 to 90 parasites/μl; moderate density = 100 to 1,000 parasites/μl, and high density = ˃ 1,000 parasites/μl.

Has been revised under the methods section.

**DISCUSSION**

1 **Comment 1:** [*The discussion section had your results repeated. Please discussion your findings in reference to malaria control in your country. Do not repeat the results.]*

**Response:** Thank you for pointing this out, we have, revised this point.

2 **Comment 2:** [*Be consistent with the use of HRP2, the discussion section has HRPII.]*

**Response:** Thank you for pointing this out, *HRPII* has been replaced by *HRP2* throughout the manuscript.
