# Supplementary material for: Assessment of the diagnostic performance of the SD Bioline Malaria antigen test for the diagnosis of malaria in the Tombel health district, Southwest region of Cameroon
Source: PLoS One. 2025 Mar 13;20(3):e0298992. doi: 10.1371/journal.pone.0298992 (PMC11906078; doi:10.1371/journal.pone.0298992)
Supplement: Attachment — Submitted filename: Response to reviewer.docx [file pone.0298992.s013.docx]

Manuscript PONE-D-23-43663R3

Response to Reviewers

Dear Dr. Enoch,

Thank you for giving me the opportunity to submit a revised draft of my manuscript titled [Assessment of the diagnostic performance of the SD Bioline malaria antigen test for the diagnosis of malaria in the Tombel health area, Southwest region of Cameroon] to *[PLOS ONE]*. We appreciate the time and effort that you and the reviewers have dedicated to providing your valuable feedback on our manuscript. We are grateful to the reviewers for their insightful comments on our paper. We have been able to incorporate changes to reflect most of the suggestions provided by the reviewers. We have highlighted the changes within the manuscript.

Here is a point-by-point response to the reviewers’ comments and concerns.

**Comments from Reviewer 1**

1 **Comment 1:** [*Please note that the plus system scale is no longer recommended by WHO. Please revise this in the methodology and show the actual parasite counts in the results section.]*

**Response:** Thank you for pointing this out. We agree with this comment. Current WHO recommendation considered the use of density scale as low density = 10 to 90 parasites/μl; moderate density = 100 to 1,000 parasites/μl, and high density = ˃ 1,000 parasites/μl which is considered as the equivalent of plus system scale: + (1 to 9 trophozoites in 100 fields); ++ (1 to 10 trophozoites in 10 fields); +++ (1 to 10 trophozoites per field); ++++ (>10 trophozoites per field) recommended by WHO. These scores were used to estimate parasite densities reported by Kosack et al [20]. However, we removed this part from the manuscript

\# Parasite densities were estimated according to parasites density levels ~~graded using the ‘plus’ system scale: + (1 to 9 trophozoites in 100 fields); ++ (1 to 10 trophozoites in 10 fields); +++ (1 to 10 trophozoites per field); ++++ (>10 trophozoites per field) recommended by WHO. These scores were~~ ~~used to estimate parasite densities~~ reported by Kosack et al [20] as low density = 10 to 90 parasites/μl; moderate density = 100 to 1,000 parasites/μl, and high density = ˃ 1,000 parasites/μl.#

to have

Parasite densities were estimated according to parasite density levels reported by Kosack et al [20] as low density = 10 to 90 parasites/μl; moderate density = 100 to 1,000 parasites/μl, and high density = ˃ 1,000 parasites/μl.

**Data Availability** The data generated or analysed during the current study are available within the manuscript and its supportive documents ~~from the corresponding author on reasonable request~~.
